# Supplementary material for: Retention of a single Cenp-C gene in different syntenic locations in the montium group of Drosophila species
Source: bioRxiv. 2025 Dec 15:2025.12.11.693522. Preprint. [Version 1] doi: 10.64898/2025.12.11.693522 (PMC12724597; doi:10.64898/2025.12.11.693522)
Supplement: Supplement 1 [file media-1.pdf]

Retention of a single *Cenp-C* gene in different syntenic locations in the *montium* group of *Drosophila* species

Rafaella F. Soares, Ching-Ho Chang, Leonardo B. Koerich, Harmit S. Malik, Gustavo C.S. Kuhn

Supplementary Material

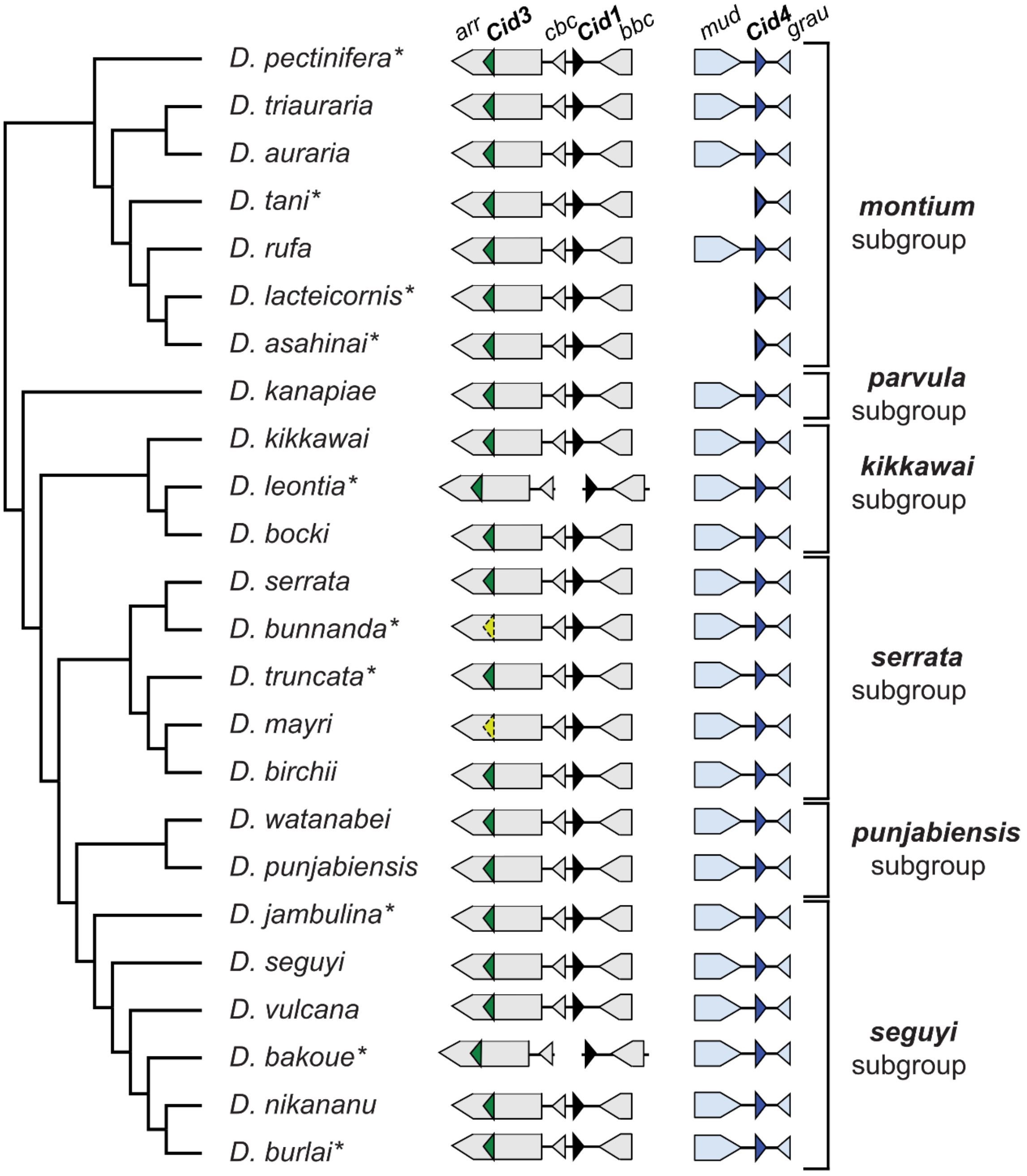

Figure S1

Phylogeny of the 24 species from the *montium* group (adapted from Conner et al., 2021) analyzed in the present work, with a schematic representation of their *Cid* paralogs. The *Cid* paralogs and their shared syntenic loci (represented by the flanking genes) are shown for all species, 14 of which were previously reported (Kursel & Malik, 2017). The additional ten species analyzed here are indicated with an asterisk. Contig breaks reflect limitations associated with short-read genome assemblies in some species.

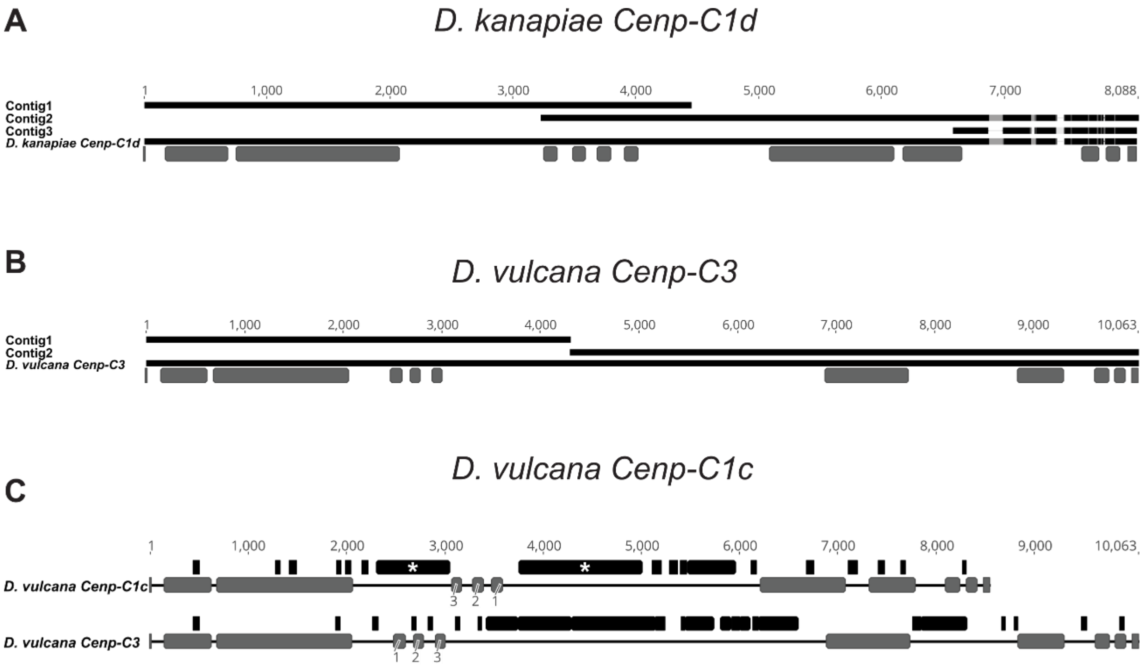

Figure S2

Predicted *Cenp-C* gene sequences for *D. kanapiae* and *D. vulcana*. (A) Alignment of the three *Cenp-C* gene fragments in *D. kanapiae* (contigs 1, 2, and 3) with the predicted *Cenp-C1d* sequence. Identical regions (black), divergent regions (gray), and gaps (white) are shown in the alignment. Gray boxes at the bottom represent exons. **(B)** Alignment of the two *Cenp-C* gene fragments in *D. vulcana* (contigs 1 and 2) with the predicted *Cenp-C3* sequence. **(C)** The *D. vulcana Cenp-1c* gene includes three exons (numbered 1-3) that appear to be inverted within a predicted intron sequence of the *Cenp-C1c* gene, flanked by transposable elements (*Helitron*-like elements, represented as a black bar with an asterisk). Black bars indicate sequences with similarity to TEs according to the RepBase database.

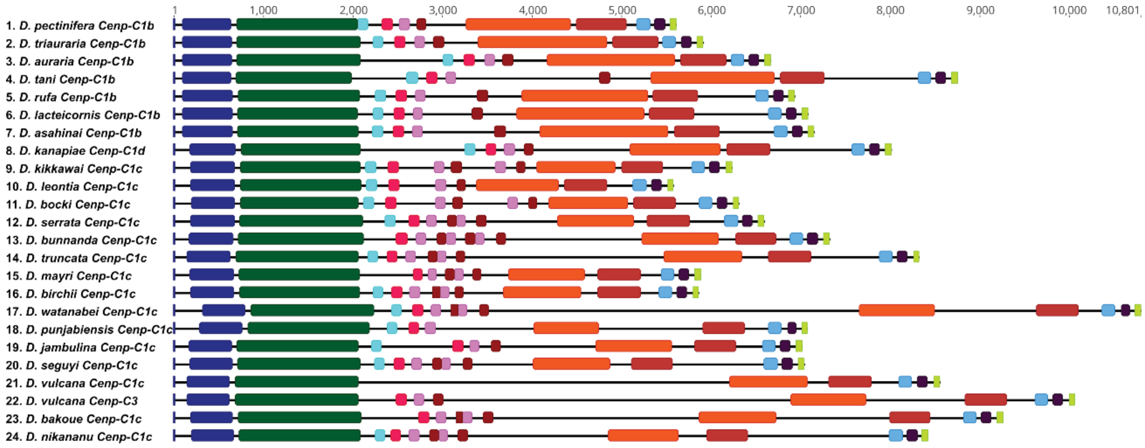

Figure S3

***Cenp-C* genes from the *montium* group.** Schematic representation of all *Cenp-C* genes, with exonic sequences shown as colored bars. Bars with the same color correspond to the equivalent sequence across *Cenp-C* genes. Exonic sequences located between the green and the orange bars in each gene represent regions that were excluded from the phylogenetic analysis because they could not be found in all species.

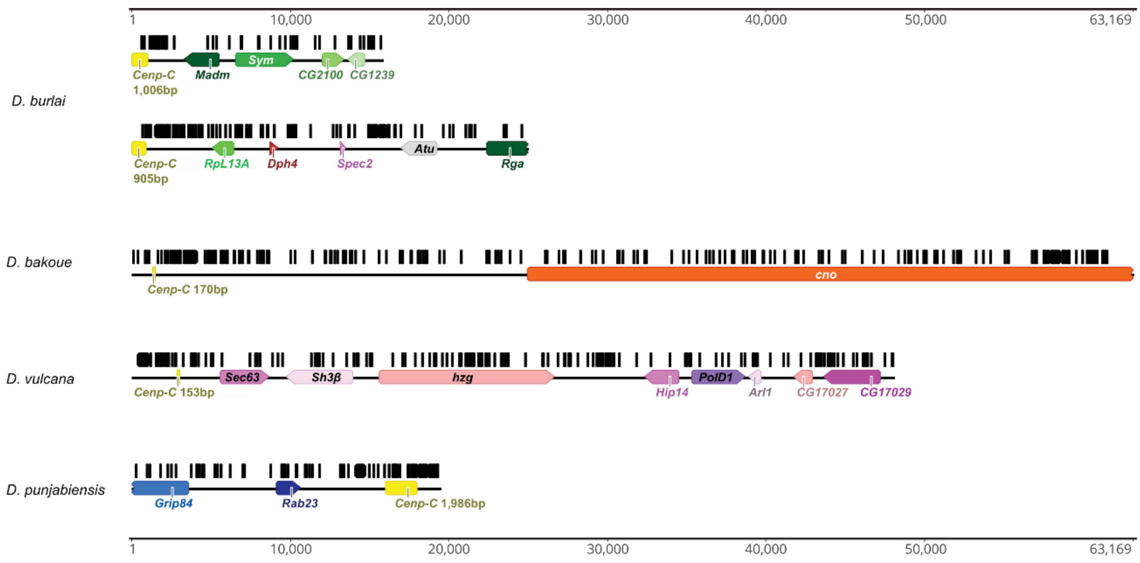

Figure S4

Additional *Cenp-C* fragments are found in distinct genomic regions across four species of the *montium* group. Genes and *Cenp-C* fragments are represented by colored bars, and intergenic sequences by black lines. Black bars indicate sequences with similarity to transposable elements, according to the RepBase database. Due to contig breaks, the genes *Rga*, *cno*, and *Grip84* are incomplete.

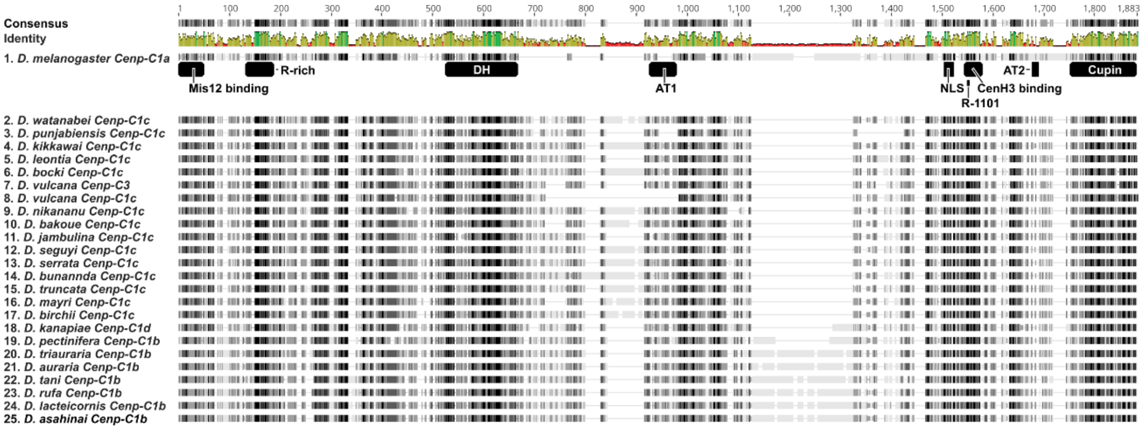

Figure S5

Alignment of Cenp-C amino acid sequences from species of the *montium* group showing the positions of the protein motifs identified in this study. The black bars represent, from left to right: Mis12 binding, R-rich, DH, AT1, NLS, CenH3 binding, AT2, and Cupin. The small bar below the CenH3 binding motif indicates the R-1101 amino acid, which is present in all Cenp-C genes from the *montium* group. Motifs AT1 and AT2 were not detected in species from the *montium* group. MEME was used to identify the Mis12 binding motif, while MAST analyses detected all the other motifs.

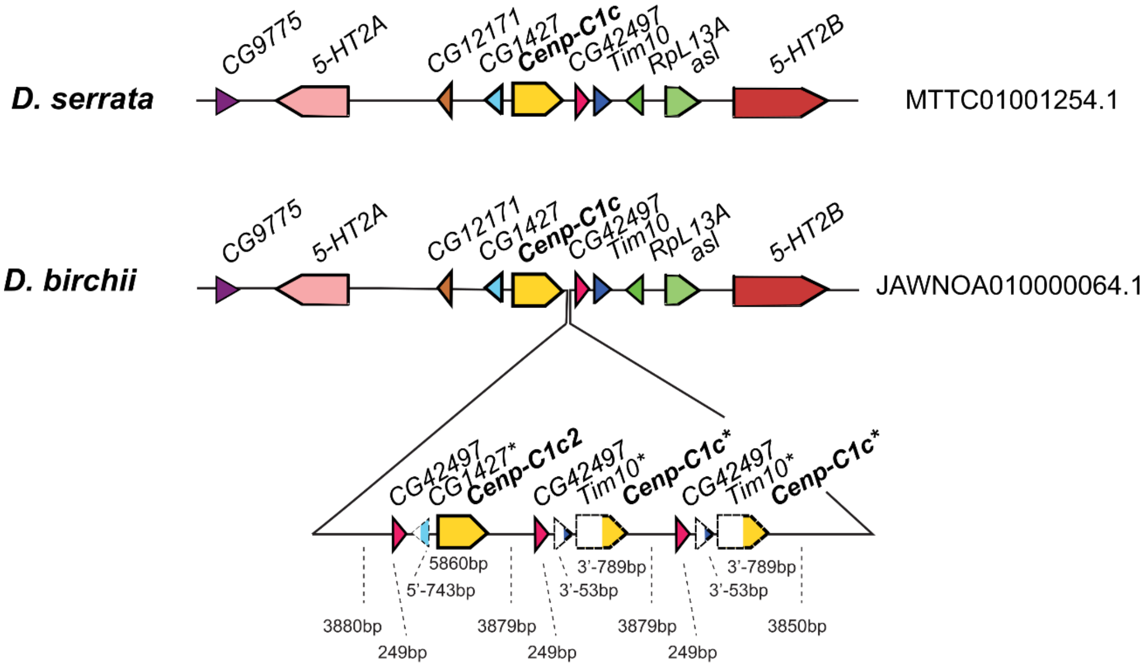

Figure S6

Tandem duplications in the *Cenp-C1c* locus of *D. birchii* revealed by long-read assembly analysis. For comparison, the corresponding locus is shown for *D. serrata*, which also belongs to the *serrata* subgroup and for which a long-read assembly was also analyzed. The tandem duplication involved both genes and intergenic sequences. Some genes were wholly duplicated, while others were partially duplicated (asterisk). For example, this locus now encodes four intact copies of *CG42497*, but only two intact copies of *Cenp-C1c*, and no additional copies of flanking genes *CG1427, Tim10,* and *RpL13A.*

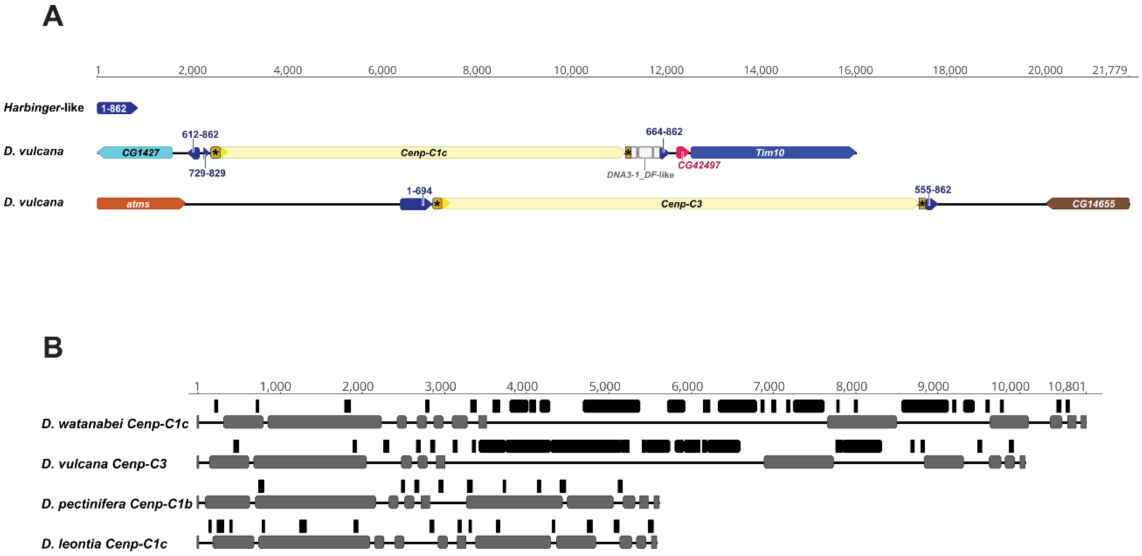

Figure S7

High density of TEs within the *Cenp-C* sequence and locus. **(A)** A repetitive sequence of approximately 862 bp is found among species of the *D. seguyi* subgroup. This sequence shows similarity to *Harbinger* elements (Repbase). Therefore, we annotate it as a *Harbinger*-like element. Segments of this Harbinger-like element flank *Cenp-C1c* and *Cenp-C3* on both sides in *D. vulcana*. The yellow bar marked with an asterisk indicates the 5′ and 3′ sequences (likely corresponding to the 5′ and 3′ UTRs) that exhibit similarity between *Cenp-C1c* and *Cenp-C3* in *D. vulcana*. The sequence between the *Cenp-C1c* 3′ UTR and the *Harbinger*-like element corresponds to a distinct DNA transposon (*DNA3-1_DF*-like element). **(B)** Enrichment of transposable elements within introns of *Cenp-C*. Schematic representation of *Cenp-C* genes from *D. watanabei* (*Cenp-C1c*), *D. vulcana* (*Cenp-C3*), *D. pectinifera* (*Cenp-C1b*), and *D. leontia* (*Cenp-C1c*). The gray bars represent exons, and the black bars represent sequences with similarity to TEs**.**

Supplementary Tables

Supplementary Table 1. Genbank Database IDs

Species	*Muller element E*	Muller element C
D. jambulina	VNJX01008392.1	VNJX01003011.1
D. watanabei	VNJS01015544.1	VNJS01016027.1
D. serrata	MTTC01001254.1	MTTC01000143.1
D. kikkawai	JARPSD010000005.1	JARPSD010000003.1
D. kanapiae	VNJM01000172.1	VNJM01002302.1
D. triauraria	JABJVT010000005.1	JABJVT010000003.1
D. melanogaster	NT_033777.3	NT_033778.4
D. ananassae	JACRYV020000138.1	JACRYV020000136.1
D. virilis	JAUDTK010000001.1	JAUDTK010000004.1
S. lebanonensis	QMEN02000020.1	QMEN02000001.1

Supplementary Table 2. Genbank Database IDs

Gene	Scaffold or genomic SRA	mRNA or transcriptomic SRA
D. pectinifera Cenp-C1b	VNKC01003598.1	Unavailable
D. triauraria Cenp-C1b	JABJVT010000005.1	SRR11780982
D. auraria Cenp-C1b	VNJW01009994.1 and **JBNRRN010000002.1**	Unavailable
D. tani Cenp-C1b	VNJO01011109.1	Unavailable
D. rufa Cenp-C1b	VNKH01000388.1 and **JAECXS010000001.1/ JAECXS010000038.1**	Unavailable
D. lateicornis Cenp-C1b	VNKF01009708.1	Unavailable
D. asahinai Cenp-C1b	VNJZ01004887.1	Unavailable
D. kanapiae Cenp-C1d	VNJM01000860.1/ VNJM01001987.1	Unavailable
D. kikkawai Cenp-C1c	AFFH02006098.1	XM_017182092.1
D. leontia Cenp-C1 c	VNKB01009439.1	Unavailable
D. bocki Cenp-C1 c	VNJY01005756.1	Unavailable
D. serrata Cenp-C1 c	MTTC01001254.1 and JAWNOG010000382.1/JAWNOG010000364.1/JAWNOG010000228.1	XM_020957133.1
D. bunnanda Cenp-C1 c	VNKE01001283.1 and **JAWNOB010000030.1/JAWNOB010000130.1/JAWNOB010000100.1**	Unavailable
D. truncata Cenp-C1 c	VNJQ01004447.1	Unavailable
D. mayri Cenp-C1 c	VNJN01008118.1	Unavailable
D. birchii Cenp-C1 c	VNKA01006487.1 and **JAWNOA010000064.1**	Unavailable
D. watanabei Cenp-C1 c	VNJS01015544.1	Unavailable
D. punjabiensis Cenp-C1 c	VNJR01008397.1	Unavailable
D. jambulina Cenp-C1 c	VNJX01008392.1 and **JAECXH010000012.1**	Unavailable
D. seguyi Cenp-C1 c	VNJU01009249.1	Unavailable
D. vulcana Cenp-C1 c	VNJP01006877.1	Unavailable
D. vulcana Cenp-C3	VNJP01005529.1/ VNJP01004469.1	Unavailable
D. bakoue Cenp-C1c	VNJL01004438.1	Unavailable
D. nikananu Cenp-C1c	VNJV01009586.1	Unavailable
D. melanogaster Cenp-C1a	NT_033777.3	NM_169228.3
D. simulnas Cenp-C1a	NIFY01000004.1	XM_016174981.1
D. sechellia Cenp-C1a	NIFZ01000004.1	XM_032720766.1
D. yakuba Cenp-C1a	JAEDAC010000005.1	XM_002096785.2
D. erecta Cenp-C1a	QMER02000001.1	XM_026983729.1
D. eugracilis Cenp-C1a	AFPQ02005741.1	SRR346729
D. elegans Cenp-C1a	WVIB01000005.1	XM_017268702.1
D. ficusphila Cenp-C1a	AFFG02008693.1	XM_017203168.1
D. takahashii Cenp-C1a	XM_017148281.1	AFFI02007576.1
D. mauritiana Cenp-C1a	XM_033307073.1	NIGA01000004.1
D. ananassae Cenp-C1a	JACRYV010000154.1	XM_001955206.3
D. persimilis Cenp-C1a	QMET02000001.1	XM_026986294.1
D. pseudoobscura Cenp-C1a	WVEN01000001.1	XM_015182067.2
D. willistoni Cenp-C1a	AAQB01009414.1	XM_023180684.1
D. mojavensis Cenp-C1a	CH933806.1	SRR6425997
D. mojavensis Cenp-C2	CH933806.1	SRR6425997
D. arizonae Cenp-C1a	SRR2070760	SRR2509638
D. arizonae Cenp-C2	SRR2070760	SRR2509638
D. navojoa Cenp-C1a	LSRL02000055.1	Unavailable
D. navojoa Cenp-C2	LSRL02000228.1	XM_018113591.2
D. buzzatii Cenp-C1a	https://dbuz.uab.cat/blast.php *D. buzzatii* Freeze 1 Scaffolds	SRR5145562/ SRR5145563

D. buzzatii Cenp-C2	https://dbuz.uab.cat/blast.php<br>*D. buzzatii* Freeze 1<br>Scaffolds	SRR5145562/<br>SRR5145563
D. seriema Cenp-C1a	ERR1976657	Unavailable
D. seriema Cenp-C2	ERR1976657	Unavailable
D. virilis Cenp-C1a	QMEO02000199.1	XM_002056576.3
D. virilis Cenp-C2	QMEO02000199.1	XM_002056451.3
D. americana Cenp-C1a	UEJX01001328.1	SRR5279019
D. americana Cenp-C2	UEJX01001683.1	SRR5279019
D. grimshawi Cenp-C1a	AAPT01020190.1	XM_001994049.2
D. grimshawi Cenp-C2	AAPT01019320.1	XM_001989754.3
D. busckii Cenp-C1a	DULD01000002.1	XM_017991761.1
D. busckii Cenp-C2	DULD01000002.1	XM_017992885.2
Phortica variegata Cenp-C1a	JXPM01003917.1	SRR1738675

NOTE: The long-read sequencing-based genome assemblies for six species are shown in bold.

Supplementary Table 3. General characteristics of the *Cenp-C* genes in species from the *montium* group.

Subgroups	Gene	nº of exons	gene length (bp)	CDS length (bp)
montium	***D. pectinifera Cenp-C1b***	10	5,610	4,386
	D. triauraria Cenp-C1b	11	5,907	4,653
	D. auraria Cenp-C1b	11	6,667	4,635
	D. tani Cenp-C1b	11	8,750	4,488
	D. rufa Cenp-C1b	11	6,933	4,599
	D. lacteicornis Cenp-C1b	11	7,078	4,590
	D. asahinai Cenp-C1b	11	7,147	4,605
parvula	***D. kanapiae Cenp-C1d***	11	8,013	4,089
kikkawai	***D. kikkawai Cenp-C1c***	13	6,229	4,152
	D. leontia Cenp-C1c	11	5,575	3,981
	D. bocki Cenp-C1c	13	6,310	4,125
serrata	***D. serrata Cenp-C1c***	12	6,591	4,152
	D. bunnanda Cenp-C1c	12	7,322	4,248
	D. truncata Cenp-C1c	12	8,324	4,131
	D. mayri Cenp-C1c	11	5,885	3,957
	D. birchii Cenp-C1c	12	5,861	4,104
punjabiensis	***D. watanabei Cenp-C1c***	12	10,801	4,068
	D. punjabiensis Cenp-C1c	10	7,076	3,657
seguyi	***D. jambulina Cenp-C1c***	11	7,015	3,867
	D. seguyi Cenp-C1c	12	7,039	4,119
	D. vulcana Cenp-C1c	7*	8,552	3,516
	D. vulcana Cenp-C3	10	10,063	3,801
	D. bakoue Cenp-C1c	11	9,264	3,981
	D. nikananu Cenp-C1c	12	8,420	4,026

NOTE: The *Cenp-C1c* from *D. vulcana* has 7 exons according to Augustus algorithm. Our analysis further revealed that *Cenp-C1c* has 10 exons (see **Supplementary Figure 2C** for more details).

Supplementary Table 4: McDonald-Kreitman tests for positive selection

Species	Gene	FET p-value	Dn	Ds	Pn	Ps	NI	α
D. melanogaster* vs *D. simulans	***Cid1*** ^{NR}	**0,015**	22	19	1	9	0,096	0,904
	CenpC ^{NR}	0,815	121	101	11	8	1,148	-0,148
**(*melanogaster* group)**	***Cal1*** ^{NR}	0,757	80	77	27	30	0,866	0,134
D. serrata* vs *D. bunnanda	***Cid1***	**0,010**	39	33	8	23	0,294	0,706
	Cid1 ^{NR}	0,088	39	34	2	8	0,218	0,782
	Cid3	0,201	54	52	16	26	0,593	0,407
	Cid3 ^{NR}	0,144	55	54	6	13	0,453	0,547
	Cid4	0,186	27	27	22	38	0,579	0,421
	Cid4 ^{NR}	**0,010**	28	29	2	15	0,138	0,862
**(*montium* group)**	***CenpC***	0,135	160	175	67	52	1,409	-0,409
	CenpC ^{NR}	0,155	162	177	12	23	0,570	0,430
	Cal1	0,811	131	137	190	189	1,051	-0,051
	Cal1 ^{NR}	0,408	142	146	62	77	0,828	0,172

NOTE. We carried out unpolarized McDonald-Kreitman tests (McDonald and Kreitman, 1991) of the *Cid, Cenp-C,* and *Cal1* genes across ~1000 *D. melanogaster* strains, using *D. simulans* as an outgroup. Since many sequenced strains were available, we ignored polymorphisms at less than 5% frequency, which are less likely to have been tested by natural selection (Fay et al. 2002); these genes are indicated with an *NR* (no-rare) superscript. In the *melanogaster-simulans* comparison, only *Cid* shows evidence of positive selection ($p<0.05$; bold), consistent with a previous study (Malik & Henikoff, 2001). We then compared the *Cid1, Cid3, Cid4, CenpC,* and *Cal1* genes from ~110 *D. serrata* sequences to those from *D. bunnanda*. However, since *D. bunnanda Cid3* is a pseudogene, we used *D. birchii Cid3* as an outgroup. We analyzed data for all polymorphisms or using comparisons that ignore rare polymorphisms (NR superscript). Our data finds evidence for positive selection in *Cid1* and *Cid4* in one of the two comparisons ($p<0.05$; bold), but no evidence for positive selection for *Cid3*, *CenpC*, or *Cal1* in either comparison.

Fay, J. C., Wyckoff, G. J., & Wu, C. I. (2002). Testing the neutral theory of molecular evolution with genomic data from Drosophila. *Nature*, *415*(6875), 1024–1026. https://doi.org/10.1038/4151024a

Malik, H. S., & Henikoff, S. (2001). Adaptive evolution of Cid, a centromere-specific histone in Drosophila. *Genetics*, *157*(3), 1293–1298. https://doi.org/10.1093/genetics/157.3.1293

McDonald, J. H., & Kreitman, M. (1991). Adaptive protein evolution at the Adh locus in Drosophila. *Nature*, *351*(6328), 652–654. https://doi.org/10.1038/351652a0
